# Sleep duration of lactating mothers and its relationship with feeding pattern, milk macronutrients and related serum factors: A combined longitudinal cohort and cross-sectional study

**DOI:** 10.3389/fnut.2022.973291

**Published:** 2022-08-30

**Authors:** Huijuan Ruan, Yajie Zhang, Qingya Tang, Xuan Zhao, Xuelin Zhao, Yi Xiang, Wei Geng, Yi Feng, Wei Cai

**Affiliations:** ^1^Department of Clinical Nutrition, Xinhua Hospital, School of Medicine, Shanghai Jiao Tong University, Shanghai, China; ^2^Shanghai Key Laboratory of Pediatric Gastroenterology and Nutrition, Shanghai, China; ^3^Shanghai Institute of Pediatric Research, Shanghai, China; ^4^Department of Pediatric Surgery, Xinhua Hospital, School of Medicine, Shanghai Jiao Tong University, Shanghai, China

**Keywords:** sleep duration, feeding pattern, milk macronutrients, human milk, lactating mothers

## Abstract

**Objective:**

Insufficient sleep is common in postpartum mothers. The main objectives of this study are to explore the sleep duration among Chinese lactating mothers and preliminarily investigate the relationship between sleep duration and feeding pattern. The secondary objectives are to investigate the relationships between sleep duration and milk macronutrients and between maternal-related indicators, including melatonin (MT), growth hormone (GH), ghrelin (GHRL), glucagon-like peptide-1 (GLP-1), prolactin (PRL), and cholecystokinin (CCK).

**Methods:**

The present study comprises a longitudinal and a cross-sectional cohort from December 2019 to December 2021. Postpartum lactating women living in Shanghai were recruited through online and offline recruitment. The subjects were included in the longitudinal cohort or cross-sectional study based on their lactation period at the time of recruitment. The longitudinal cohort included a total of 115 mothers. Human milk and feeding pattern were measured and collected at 2–4 months and 5–7 months postpartum. At four predetermined follow-up time points, data on sleep duration was collected (at the time of recruitment, 2–4 months postpartum, 5–7 months postpartum, and 12–17 months postpartum). The cross-sectional study included 35 lactating mothers (2–12 months postpartum) who reported their sleep duration and provided blood samples. Mid-infrared spectroscopy (MIRS) method was used to analyze the macronutrients of breast milk, while MT, GH, GHRL, GLP-1, PRL, and CCK in maternal blood were determined by ELISA.

**Results:**

The maternal sleep duration before pregnancy was 8.14 ± 1.18 h/d (*n* = 115), 7.27 ± 1.31 h/d (*n* = 113) for 2–4 months postpartum, 7.02 ± 1.05 h/d (*n* = 105) for 5–7 months postpartum, and 7.45 ± 1.05 h/d (*n* = 115) for 12–17 months postpartum. The incidence of insufficient sleep (<7 h/d) before pregnancy (12.17%) was significantly less than at any follow-up time after delivery (vs. 2–4 months postpartum, χ^2^ = 10.101, *p* = 0.001; vs. 5–7 months postpartum, χ^2^ = 15.281, *p* < 0.0001; vs. 12–17 months postpartum, χ^2^ = 6.426, *p* = 0.011). The percentage of insufficient maternal sleep was highest at 5–7 months postpartum (34.29%). No significant difference was found between the incidence of insufficient sleep at 5–7 months postpartum, 2–4 months postpartum (29.20%, χ^2^ = 0.650, *p* = 0.420), and 12–17 months postpartum (25.22%, χ^2^ = 2.168, *p* = 0.141). At 2–4 months postpartum, the frequency of formula feeding per day is related to reduced maternal sleep duration (Standardization coefficient β = −0.265, *p* = 0.005, Adjusted R^2^ = 0.061). At 2–4 months and 5–7 months postpartum, the relationship between macronutrients in breast milk and the mother's sleep duration was insignificant (all *p* > 0.05). Other than the positive correlation found between maternal GHRL and sleep duration (*r* = 0.3661, *p* = 0.0305), no significant relationship was observed between sleep duration and other indexes (all *p* > 0.05).

**Conclusions:**

Postpartum mothers generally sleep less, but there is no correlation between insufficient sleep and the macronutrient content of breast milk. Formula feeding may be related to the mother's sleep loss, while breastfeeding (especially direct breastfeeding) may be related to increased maternal sleep duration. The findings suggest that sleep duration is related to maternal serum GHRL. More high-quality studies are needed to clarify the mechanism of these findings and provide a solid theoretical basis and support references for breastfeeding.

## Introduction

The perinatal period means a series of changes for mothers. Mothers need to feed and care for their babies frequently during lactation, causing sleep interruptions and reducing overall sleep quality and quantity (1). From late pregnancy to a few years after delivery, women are vulnerable to high incidence rates and long periods of sleep disorders (1–3).

Previous research has found that women average <7 h of sleep per day over the postpartum period, and more than half of women report sleep difficulties in the postpartum period (4, 5). Lack of sleep during postpartum can have numerous detrimental effects, including increased depressive or anxious symptomatology, development of postpartum depression (PPD), fatigue, and unfavorable impact on lower milk volume (1, 3, 6–10). The Akershus Birth Cohort Study concluded that mothers' insomnia prevalence in the 2nd year of postpartum was high (41%) and the average sleep duration was 6 h 52 min (11). In a longitudinal observational study in China, the incidence of poor sleep quality from 42 days postpartum to 3 years postpartum was 31.7 to 75.1%, of which 42 days postpartum was the peak, showing an inverted U-shaped curve as a whole (12).

Postpartum women also experience altered sleep patterns that may lead to sleep disturbances. The most common reasons for sleep disturbances are related to newborn sleep and feeding patterns (13). Based on the limited research exploring why mothers stop breastfeeding, fatigue associated with breastfeeding is one of the major factors (4, 5, 14). Women may believe formula feeding affords them more opportunity to rest. Indeed, qualitative research indicates that healthcare professionals and new mothers may request formula feeding due to the fatigue and tiredness that is often displayed in breastfeeding women (4, 15). This reasoning indicates a potential association between less sleep for women postpartum and breastfeeding behavior.

In contrast, other studies have found sleep advantages among breastfeeding women. Breastfed mothers are more likely to get deeper sleep and better sleep quality than formula-fed mothers (4, 16). Therefore, given inconsistent results and insufficient data on sleep and feeding patterns, especially among lactating women in China, further research is needed to understand better the potential links between sleep duration and feeding patterns.

Given that insufficient postpartum sleep is common, more research needs to focus on exploring the effects of sleep deprivation, including lactation (including changes in macronutrients in breast milk) and the condition of mothers themselves. Short-term and long-term insufficient sleep duration may adversely affect people's health and wellbeing (17–22). Lack of sleep can lead to changes in human hormone secretion. For instance, obesity can be related to insufficient sleep (23). A dose-response effect is present when the effect increases with sleep duration, and appetite markers play a significant role (24, 25). Reduced sleep duration and quality, as well as circadian desynchronization of the sleep–wake cycle resulting in increased hepatic glucose production and decreased peripheral glucose uptake *via* neuroendocrine efferences and modulation of autonomous nervous system activity (eg, in muscles), changes in pancreatic α-cell and β-cell function, increased stress axis activity (eg., enhanced adrenal cortisol and catecholamine release), and change in the secretion of appetite-regulating hormone (eg., ghrelin, leptin) from the gastrointestinal tract and adipose tissue, promoting food intake (25). Sleep also affects melatonin (MT); low MT levels are associated with improved depression mood in postpartum mothers (26). Prolactin (PRL) is related to milk secretion. So far, little is known about the sleep duration of lactating mothers and the effects of insufficient sleep on lactation and associated humoral factors. In addition, a study found that shortened postpartum sleep duration may be related to less milk secretion (3). However, the study did not explore the relationship between sleep and milk macronutrients.

The main objectives of this study are to explore the sleep duration among Chinese lactating mothers and preliminarily investigate the relationship between sleep duration and feeding pattern. The secondary objectives are to investigate the relationships between sleep duration and milk macronutrients and between maternal-related indicators, including MT, growth hormone (GH), ghrelin (GHRL), glucagon-like peptide-1 (GLP-1), PRL, and cholecystokinin (CCK). The results of this study can be used to promote optimal maternal and infant health and support breastfeeding.

## Materials and methods

### Objects of study

The dataset was obtained from a maternal and infant nutrition study in the Xinhua Hospital (MINCXH) involving a longitudinal cohort and cross-sectional study. Postpartum lactating women living in Shanghai were recruited for this study through online and offline recruitment. They were included into the cross-sectional or longitudinal cohort based on the recruitment inclusion and exclusion criteria and the subjects' wishes. Before starting the study, we used a sample size calculator (http://powerandsamplesize.com/Calculators/Compare-k-Means/1-Way-ANOVA-Pairwise, power = 0.80; α = 0.05; G = 3) and the recommendations from previous studies to determine the number of people that would have to be recruited (27–29). For the initial step, we identified 123 mothers in the longitudinal cohort, of which 115 were eventually included. Follow-ups were conducted 12–17 months after delivery to monitor the maternal sleep duration. The overall recruitment time of the subjects was from December 2019 to December 2020. The last follow-up was conducted from February 2021 to December 2021. Therefore, this study began in December 2019 and ended in December 2021. Additional information on the participant population is available in the previous studies (27, 28). The inclusion and exclusion criteria for the longitudinal cohort and the cross-sectional study are as follows:

### Longitudinal cohort

#### Inclusion criteria

Chinese mothers who have been breastfeeding; living in Shanghai for more than six months; lactating within 14 days postpartum at the time of recruitment (28).

#### Exclusion criteria

Lack of breast milk leading to the cessation of breastfeeding; inability or unwillingness to provide milk; unable to communicate due to language barriers or mental health problems; severe medical condition(s) requiring medication; lost contact before collecting breast milk; failure to collect breast milk as needed; failure to store breast milk as required (28).

#### Follow-up strategy

Human milk and feeding pattern information were collected within 2–4 months postpartum and 5–7 months postpartum; sleep duration information was collected at the time of recruitment for the information before pregnancy. During the follow-up, the mother's sleep duration was collected at 2–4 months postpartum, 5–7 months postpartum and 12–17 months postpartum.

### Cross-sectional study

#### Inclusion criteria

Chinese mothers who have been breastfeeding; living in Shanghai for more than six months; lactating after 14 days postpartum at the time of recruitment (28).

#### Exclusion criteria

Lack of breast milk leading to the cessation of breastfeeding; inability or unwillingness to provide milk; unable to communicate due to language barriers or mental health problems; with severe medical condition(s) requiring medication; lost contact before collecting breast milk; failure to collect breast milk as required; failure to store breast milk as required (28).

#### Follow-up strategy

Maternal blood, and sleep duration information were collected once during the mature milk period (2–12 months postpartum).

**Figure 1:**
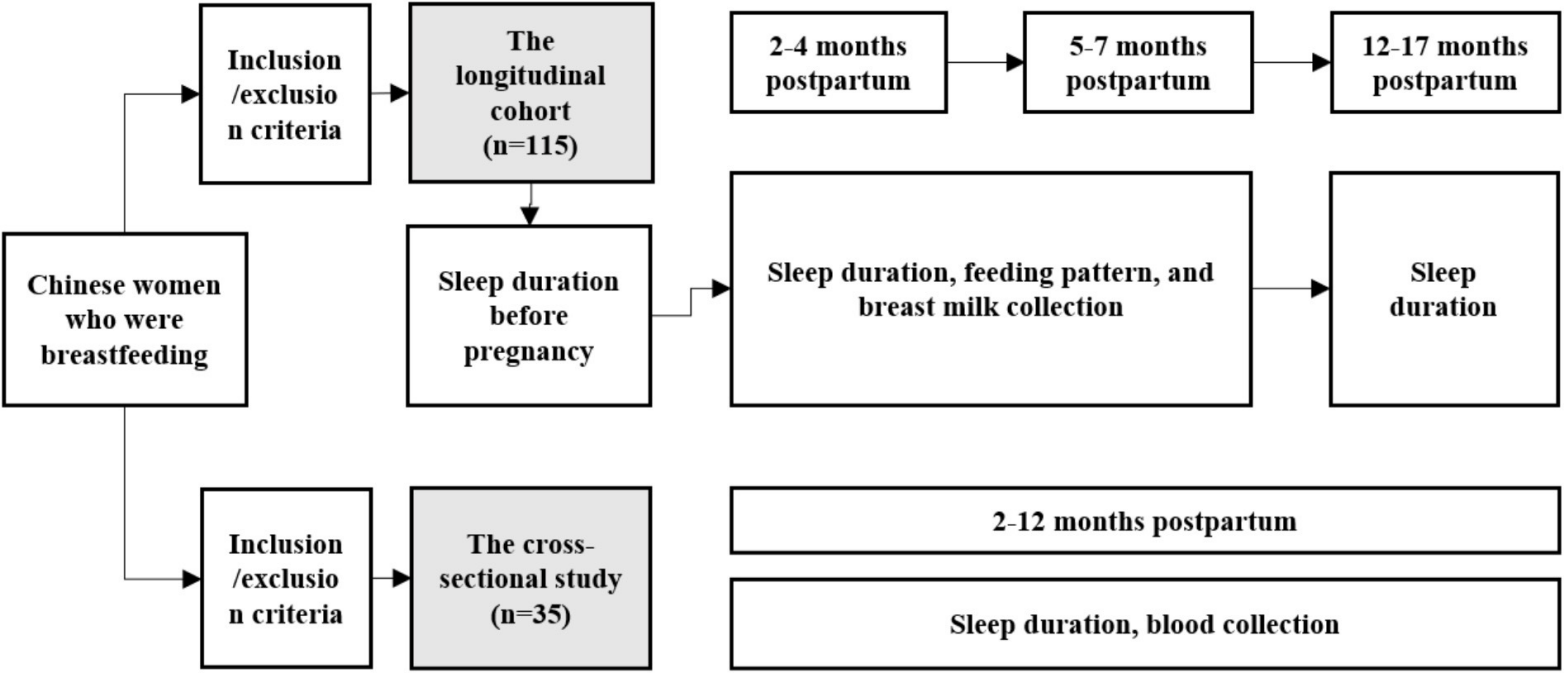
Study design flow chart.

This study was approved by the hospital's Ethics Committee with approval number XHEC-C-2020-081, and written informed consents have been obtained. The flowchart is shown in Figure 1.

### Breast milk collection

After fasting for more than 8 h and before feeding their babies, the mothers collected their breast milk each morning using an electric breast pump, emptying milk from one side of the breast. After mixing well, at least 5 ml of milk was taken as test sample, put into a unified breast milk collection bag, and refrigerated at −20°C for temporary storage. Arrangements were made to transfer the test milk samples through the cold chain. The mothers were contacted for recollection in case of deterioration, impurities, container rupture, or other sample contamination. Following the milk transfer to the study group at the Xinhua Hospital, it was stored in a lab refrigerator at −80°C for analysis. Breast milk collection, transportation, and storage have been discussed in previous studies (30, 31). More details about breast milk collection can be found in the previous works of literatures (27, 28).

### Analysis of milk macronutrients

We used a milk analyzer (BETTERREN Co., HMIR-05, SH, CHINA) based on the mid-infrared spectroscopy (MIRS) method to evaluate the milk macronutrients. The MIR-based analyzer has been widely used, and its accuracy has been verified in previous studies (32–35). The analysis process was performed following the instrument operating instructions.

### Mother's sleep duration and other essential information

The mother self-reported sleep duration, and the results were accurate to 0.1 h. In the longitudinal cohort, the mother's pre-pregnancy sleep duration was collected at the first postpartum follow-up, and the sleep duration at each stage was collected at corresponding follow-up points. For the first three follow-up points (at the time of recruitment, 2–4 months postpartum, and 5–7 months postpartum), sleep information was collected using a questionnaire, while the information for the last follow-up point (12–17 months postpartum) was collected *via* WeChat or telephone interview. At the first follow-up (after recruitment), the mother was asked about the average daily sleep duration before pregnancy. Mothers were asked to provide sleep duration in the past month at the remaining three follow-up time points (2–4 months postpartum, 5–7 months postpartum, and 12–17 months postpartum).

In the cross-sectional study, sleep information was provided using same questionnaires, in which mothers (2–12 months postpartum) were asked to provide sleep duration in the past month. If the daily sleep duration differed, the average sleep for 1 week was used and included in the statistical analysis.

The maternal height, infant birth weight, number of deliveries and gestational age were self-reported at the first postpartum follow-up. A nutritionist with 13 years of work experience preliminarily collected and assessed the quality of questionnaire responses (including sleep duration and feeding patterns). If important information were not filled in or some data were in doubt, the mother was immediately contacted through Wechat or telephone for verification.

### Feeding pattern

In the longitudinal cohort, the feeding patterns of infants were recorded and analyzed 2–4 months postpartum and 5–7 months postpartum. Using the questionnaires, the mothers recorded the infant feeding pattern (e.g., breastfeeding, formula feeding and mixed feeding) within 1 week at the corresponding follow-up stage. If the mother is exclusively breastfeeding, the questionnaire records whether the baby is fed directly or indirectly through bottles or other containers. In case of formula feeding, the frequency and the amount of each feeding are to be recorded. For mixed feeding, both information related to breastfeeding and formula feeding had to be documented. In addition, the following parameters for feeding patterns were obtained:

Total feeding (TF) (times) refers to the frequency of total feeding per day (including breastfeeding and formula feeding); direct breastfeeding (DB) (times) refers to the frequency of direct breastfeeding per day; indirect breastfeeding (IB) (times) refers to the frequency of breastfeeding through bottles or other containers per day; formula feeding (FF) (times) refers to the frequency of formula feeding per day; bottle feeding (BF) (times) refers to the frequency of bottle feeding per day (including breastmilk feeding through bottles and formula feeding); direct breastfeeding (DB) (%) refers to the percentage of the frequency of direct breastfeeding in the frequency of total feeding per day; and human milk (%) refers to the percentage of breastmilk feeding per day. A nutritionist also checked the above informations after being collected.

### Collection and analysis of blood samples

In the cross-sectional study, thirty-five mothers agreed to the blood collection and signed the informed consent forms according to the principle of voluntariness. The mother's blood was collected in the hospital at 8–10 a.m. after fasting for at least 8 h. The blood samples were collected by qualified nurses and were subjected to standing and centrifugation. Then, the analyzed plasma was sub-packed and frozen at −18°C until detection. MT, GH, GHRL, GLP-1, PRL and CCK were determined using ELISA according to the manufacturer's instructions (X-Y Biotechnology Co., Ltd, Shanghai, China, Elabscience Biotechnology Co., Ltd, Wuhan, China). The pre-experiment was first carried out, and the formal experiment was conducted twice, taking the average value as the final test result. The actual (raw) experimental results have been saved in Excel form.

### Statistical analysis

Statistical analysis was performed using SPSS Statistics 25.0 (IBM Co., Armonk, NY, USA). Continuous variables were presented as mean ± SD. One-way analysis of variance (ANOVA) was used to compare the difference between groups, while the least square distance (LSD) method was used to compare data between groups. Chi-square analysis was used to compare the incidence of sleep loss at different periods. Bonferroni correction was used to adjust the results of multiple comparisons. The formula of Bonferroni correction is p (1/*n*), where p is the actual threshold (0.05), and n is the total number of tests. Pearson's correlation analysis was used to assess the correlation between sleep duration and other indicators, while partial correlation analysis was used after controlling variables. Multiple linear regression was used further to analyze the relationship between sleep duration and feeding patterns. Collinearity diagnostics were carried out according to the variance inflation factor (VIF) (36). R-language and Prism 9.0 were used to generate the figures, and the *p-*value <0.05 was used as gauge to indicate statistical significance.

## Results

### Characteristics of all the subjects

In the longitudinal cohort, 115 mothers with an average age of 31.12 ± 4.03 were finally included in the study. Their average height were 161.54 ± 5.65 cm. Their infant birth weight were 3.20 ± 0.54 kg, and their average number of deliveries were 1.37 ± 0.624. The average duration of follow-up was 10.17 ± 3.05 days, 80.66 ± 21.18 days, 164.75 ± 14.92 days, and 429.43 ± 37.98 days, respectively. Among them, 111 completed one-milk collection, and 106 completed two-milk collection. A total of 448 questionnaires containing general and sleep information and 217 milk samples were collected at the various lactation stages. Statistical data on the feeding patterns and milk macronutrients are summarized in Table 1.

**Table 1:** Feeding patterns and milk macronutrients of the subjects in the longitudinal cohort.

	**Month 2–4 p.p**	**Month 5–7 p.p**	***p*-value**
**Feeding patterns(** ***n*** **)**	113	105	
TF (times)	8.6 ± 2.4	6.8 ± 1.3	0.001
DB (times)	5.8 ± 3.0	3.9 ± 2.7	<0.0001
IB (times)	1.5 ± 2.3	1.7 ± 1.9	<0.0001
FF (times)	1.3 ± 2.1	1.2 ± 1.8	<0.0001
BF (times)	2.8 ± 3.1	2.9 ± 2.4	<0.0001
DB (%)	68.5 ± 35.6	55.1 ± 36.5	<0.0001
Human milk (%)	87.1 ± 20.4	83.3 ± 24.6	<0.0001
Direct breastfeeding/human milk (%)	76.7 ± 34.8	64.1 ± 37.4	<0.0001
Milk composition (*n*)	111	106	-
Protein (g/dl)	1.35 ± 0.08	1.34 ± 0.08	0.792
Fat (g/dl)	3.34 ± 0.95	3.16 ± 0.98	0.145
Lactose (g/dl)	7.89 ± 0.46	8.09 ± 0.48	0.005
Energy (kj/dl)	293.50 ± 34.02	289.56 ± 34.51	0.318

Repeated measurement ANOVA was used. P-value refers to the statistical value of fixed effect analyzed by ANOVA.

TF (times), total feeding (times) refers to the frequency of total feeding per day, including breastfeeding and formula feeding.

DB (times), direct breastfeeding (times) refers to the frequency of direct breastfeeding per day.

IB (times), indirect brestfeeding (times) refers to the frequency of indirect breastfeeding per day, generally referring to feeding the mother's milk through bottles or other containers.

FF (times), formula feeding (times) refers to the frequency of formula feeding per day.

BF (times), bottle feeding (times) refers to the frequency of bottle feeding per day, including feeding the mother's milk through bottles and formula feeding.

DB (%), direct breastfeeding (%) refers to the percentage of the frequency of direct breastfeeding in the frequency of total feeding per day.

human milk (%), the percentage of human milk breastfeeding in the frequency of total feeding per day.

In the cross-sectional study, 35 questionnaires were collected among the respondents [31.33 ± 3.29 years old, 179.32 ± 106.30 days postpartum], and all respondents were tested for serum indicators.

### Maternal sleep duration from pre-pregnancy to postpartum

**Figure 2:**
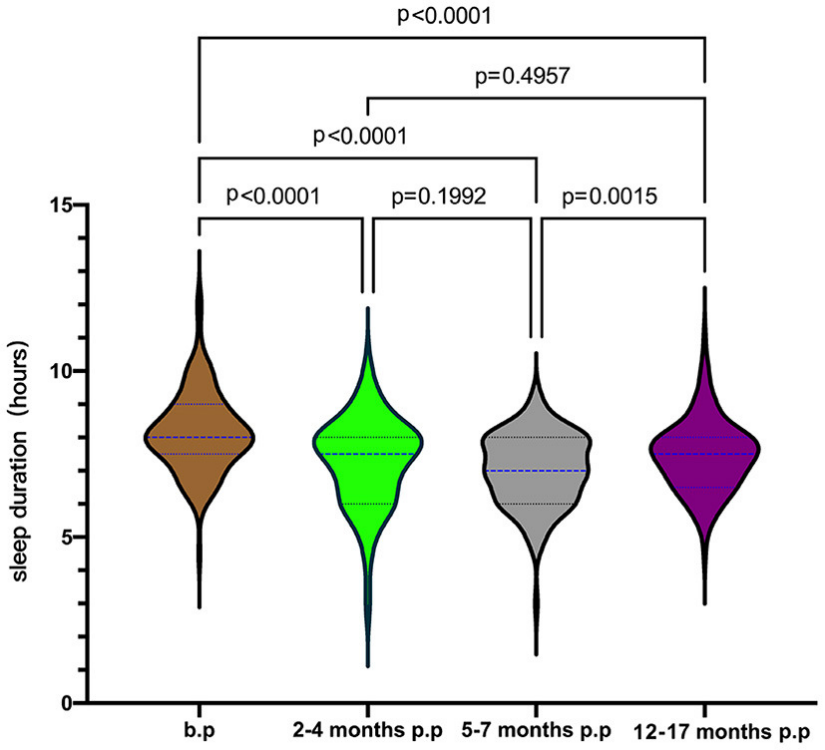
Maternal sleep duration from pre-pregnancy to postpartum. b.p, before pregnancy; p.p, postpartum.

In the longitudinal cohort, the maternal sleep duration before pregnancy was 8.14 ± 1.18 h/d (*n* = 115), 7.27 ± 1.31 h/d (*n* = 113) for 2-4 months postpartum, 7.02 ± 1.05 h/d (*n* = 105) for 5–7 months postpartum, and 7.45 ± 1.05 h/d (*n* = 115) for 12–17 months postpartum. As shown in Figure 2, we found significant differences in sleep duration at different stages (*p* < 0.05). After Bonferroni correction, there were still significant differences between groups at the level of adjusted *P* = 0.0083. The sleep duration at each postpartum stage was shorter than before pregnancy, reaching the lowest at 5–7 months postpartum.

**Figure 3:**
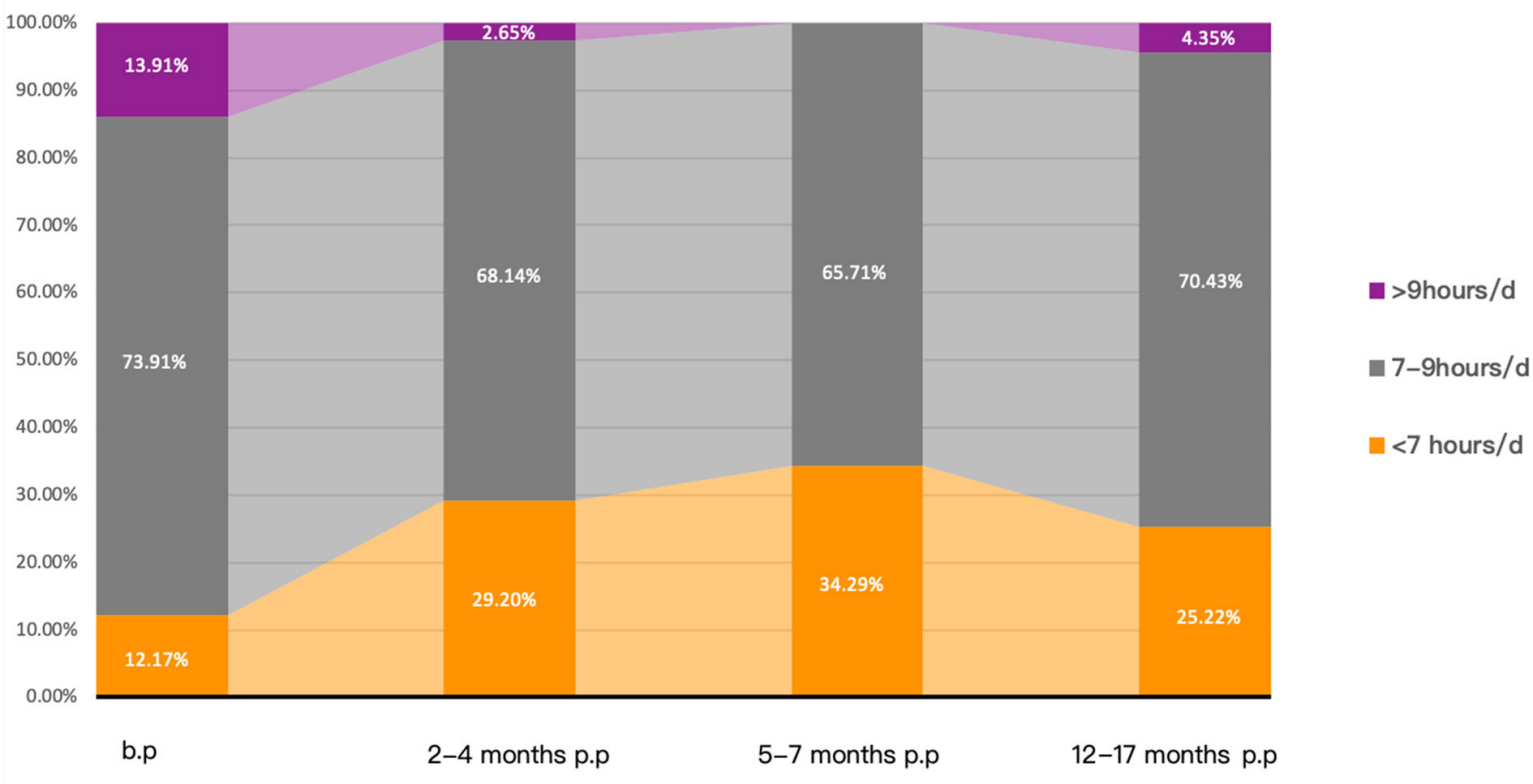
Percentage distribution of sleep duration. b.p, before pregnancy; p.p, postpartum.

Based on the recommendations of the American Sleep Foundation, we classified the sleep duration of the subjects into three groups: (1) <7 h/d, (2) 7-9 h/d, and (3) >9 h/d (37). The proportion of pregnant women with insufficient sleep (<7 h/d) before pregnancy is 12.17% (14/115), 29.20% (33/113) at 2–4 months of postpartum, 34.29% (36/105) at 5–7 months of postpartum, and 25.22% (29/115) at 12–17 months of postpartum, as shown in Figure 3. In the chi-square analysis, the incidence of insufficient sleep (<7 h/d) before pregnancy (12.17%) was significantly less than at any follow-up time after delivery (vs. 2–4 months p.p, χ^2^ = 10.101, *p* = 0.001; vs. 5–7 months p.p, χ^2^ = 15.281, *p* < 0.0001; vs. 12–17 months p.p, χ^2^ = 6.426, *p* = 0.011). The percentage of insufficient maternal sleep was highest at 5–7 months postpartum (34.29%). No significant difference was found between the incidence of insufficient sleep at 5–7 months postpartum, 2–4 months postpartum (29.20%, χ^2^ = 0.650, *p* = 0.420), and 12–17 months postpartum (25.22%, χ^2^ = 2.168, *p* = 0.141).

### Relationship between sleep duration and feeding pattern

**Figure 4:**
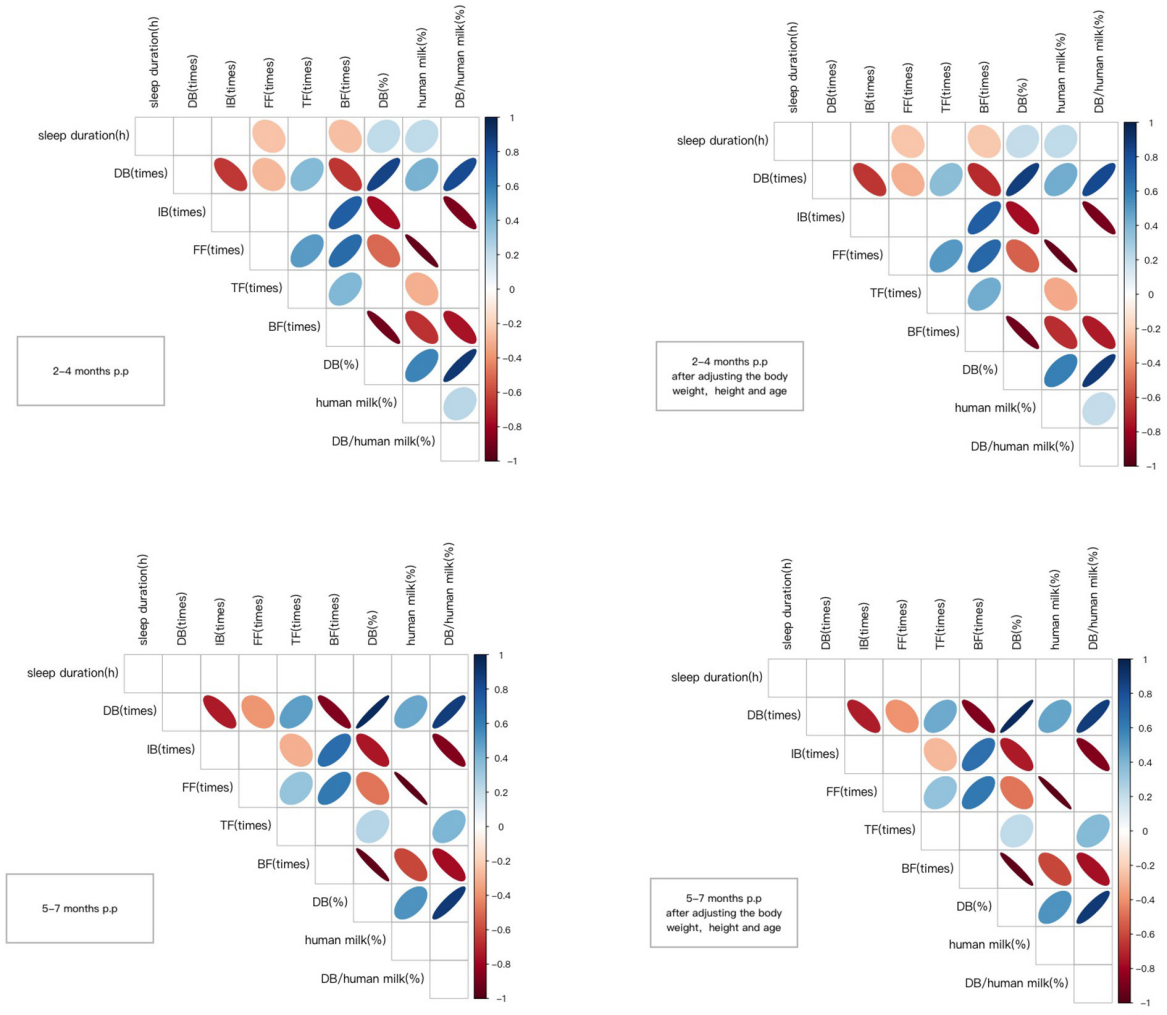
Correlation coefficients between the variables. Only significant correlations are shown (*p* < 0.05) either in blue (positive) or in red (negative). The Color intensity and size of the ellipse are proportional to the correlation coefficients. DB (times), refers to the frequency of direct breastfeeding per day; IB (times), refers to the frequency of indirect breastfeeding per day, generally referring to feeding the mother's milk through bottles or other containers; FF (times), refers to the frequency of formula feeding per day; TF (times), refers to the frequency of total feeding per day, including breastfeeding and formula feeding; BF (times), refers to the frequency of bottle feeding per day, including feeding the mother's milk through bottles and formula feeding; DB (%), refers to the percentage of the frequency of direct breastfeeding in the frequency of total feeding per day; human milk (%), the percentage of the frequency of human milk breastfeeding in the frequency of total feeding per day.

In the longitudinal cohort, the sleep duration and feeding patterns at 2–4 months and 5–7 months postpartum were analyzed using correlation analysis. We found that at 2–4 months postpartum, the mother's sleep duration was negatively correlated with FF (times) and BF (times). Maternal sleep duration was positively correlated with DB (%) and human milk (%). After adjusting the basic information of the mothers, including height, weight and age, the correlation was still statistically significant. In comparison, no significant relationship was found between maternal sleep duration and feeding pattern at 5–7 months postpartum. The detailed summary of results is presented in Figure 4.

We incorporated candidate indexes into the multiple linear regression model to explore the relationship between sleep duration and feeding pattern. DB (times), IB (times), FF (times), TF (times), BF (times), DB (%), human milk (%) were considered as candidate indicators. After collinearity diagnostics (VIF <10), DB (times), IB (times), and FF (times) were included in the linear regression. The FF (times) for 2–4 months postpartum was successfully entered into the model using stepwise regression, whereas no index was included for 5–7 months postpartum. Table 2 shows the specific statistics.

**Table 2:** Statistics of multiple linear regression.

	**Dependent variables**	**Factors**	**Standardization coefficient β**	***p***	***R***	***R^2^***	***Adjusted R^2^***
Month 2–4 p.p	Sleep duration (h)	FF (times)	−0.265	0.005	0.265	0.070	0.061
Month 5–7 p.p	Sleep duration (h)	–	–	–	–	–	–

p.p, postpartum; FF (times), refers to the frequency of formula feeding per day.

### Relationship between sleep duration and milk macronutrients

**Figure 5:**
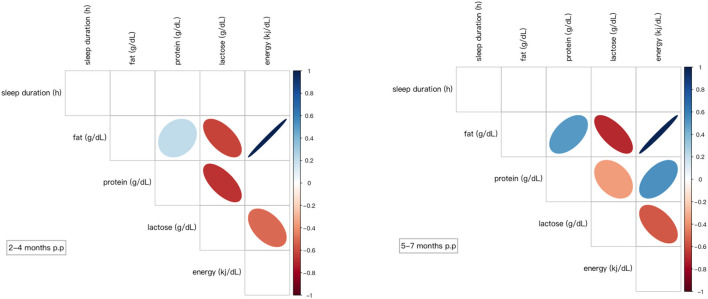
Correlation coefficients between the variables. Only significant correlations are shown (*p* < 0.05) either in blue (positive) or in red (negative). Color intensity and size of the ellipse were proportional to the correlation coefficients. p.p, postpartum.

In the longitudinal cohort, there was no significant relationship between macronutrients in breast milk and maternal sleep duration, whether at 2–4 months postpartum or 5–7 months postpartum, as shown in Figure 5.

### Maternal sleep duration and related hormones

**Figure 6:**
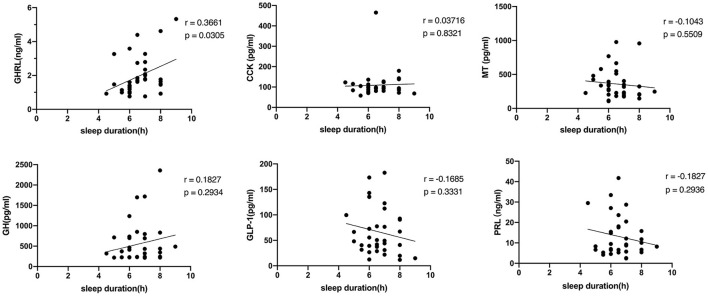
The relationship between the serum indicators and sleep duration. GHRL, ghrelin; CCK, cholecystokinin; MT, melatonin; GH, growth hormone; GLP-1, glucagon-like peptide-1; PRL, prolactin.

Thirty-five questionnaires were collected among the respondents in the cross-sectional study, and all were tested for serum indicators. The average sleep duration of these mothers is 6.61 ± 1.00 h per day, with a maximum of 9 h and a minimum of 4.5 h. MT, GH, GHRL, GLP-1, PRL, and CCK in maternal blood were 357.32 ± 216.38 pg/ml, 552.56 ± 501.31 pg/ml, 1.97 ± 1.13ng/ml, 66.69 ± 46.09pg/ml, 13.00 ± 9.61 ng/ml, and 109.32 ± 66.78 pg/ml respectively. Besides the positive correlation between GHRL and sleep duration (*r* = 0.3661, *p* = 0.0305), no relationship was found between sleep duration and other indexes, as shown in Figure 6.

## Discussion

Insufficient sleep is ubiquitous worldwide (38, 39) and is particularly serious among lactating women. In this study, we first investigated the sleep duration of lactating mothers and explored the relationship between sleep duration and feeding patterns. Secondly, we analyzed the relationship between lactating mothers' sleep duration and milk macronutrients. Finally, we observed the impact of sleep duration on related serum factors. The study's results provide new perspectives on sleep among lactating mothers and a unique theoretical basis to support breastfeeding.

Our results show that mothers have the least amount of sleep at 5–7 months postpartum and that maternal sleep length does not return to pre-pregnancy levels even 12–17 months after childbirth. The percentage of mothers experiencing insufficient sleep postpartum was significantly higher than before pregnancy. Similar results were found in a study, which concluded that exclusive breastfeeding is associated with reduced maternal sleep duration (average 7.08 h daily) (2). Another study found that breastfeeding was associated with a slight decrease in maternal sleep satisfaction, with both parents experiencing long-term effects on sleep due to the arrival of new family members (40).

Public opinion and even some professionals believe that mothers lack sleep because they need to feed their babies and that using formula would help improve the situation (2, 4, 15, 41). However, other studies hold different views (4, 16). This study explored the effects of different feeding patterns on maternal sleep duration. We found that breastfeeding during 2–4 months postpartum helps reduce insufficient maternal sleep and that formula feeding may further aggravate the reduction of maternal sleep duration. Similar results were observed in another study, which found that breastfeeding mothers had better sleep quality than formula-feeding mothers (42). Another study concluded that breastfeeding women slept an average of 2.6 h more per day in early postpartum than formula-feeding women (43). One possible reason is that breast milk MT, which is not present in the formula, helps promotes infant sleep; if the baby goes to sleep quickly, the mother can also take her rest (44). Interestingly, our results show that the relationship between infant feeding pattern and sleep duration disappeared at 5–7 months postpartum. At all follow-up points in our study, mothers had the shortest sleep duration at 5–7 months after giving birth. One possible reason is that most mothers in our research ended their maternity leave and returned to work around 5 months postpartum. Due to the relatively fixed commuting time, mothers who require lactation at night cannot compensate for lost sleep, so the overall sleep duration is reduced. Therefore, returning to work may have considerable effect on the relationship between feeding patterns and sleep duration.

Although a few previous studies have discussed the relationship between sleep duration and lactation, the relationship between sleep duration (especially insufficient sleep) and breast milk macronutrients is still unclear. When analyzing the relationship between sleep duration and milk macronutrients, we found no correlation between macronutrients in breast milk and sleep duration. The results suggest that the decrease in postpartum sleep has no significant effect on the macronutrient content of breast milk. Insufficient sleep can harm humans, but fortunately, it does not seem to affect the macronutrients in breast milk. This reasoning suggests that breast milk composition may be less affected by adverse lifestyle (such as lack of sleep) to some extent, which further supports that breast milk is undoubtedly the best food for infants. This finding has not been reported in the previous literature, and its mechanism needs to be further explored. Future research can further explore the relationship between sleep duration and other breast milk components, such as active proteins.

According to previous reports, sleep duration is likely related to several hormones. Our study preliminarily explored the relationship between sleep duration among lactating mothers and some humoral factors i.e., GHRL, CCK, MT, GH, GLP-1, and PRL. GHRL plays an important role in metabolism and appetite regulation and has been found to participate in the energy balance during sleep (45). CCK is a hormone produced in the small intestine and is related to satiety (46). MT is a well-known hormone closely associated with sleep (47). GH has numerous biological functions, including promoting growth, energy mobilization, gonadal development, and appetite (48). GLP-1 is an endogenous gut hormone and a key regulator in maintaining glucose homeostasis by stimulating insulin secretion (49). PRL is a hormone closely related to lactation behavior (50). Only the positive correlation between GHRL and sleep duration was significant in this study. A clinical study found that the GHRL level in patients with chronic insomnia was significantly reduced (51). In another clinical study, plasma GHRL levels are elevated after one night of total sleep deprivation (52). The results of these studies on GHRL and sleep suggest that human plasma GHRL levels can have diametrically opposite reactions in the case of acute and chronic sleep deprivation. In the present study, the linear relationship between plasma GHRL and sleep duration of lactating mothers is consistent with that of people with chronic insomnia (51). More research is needed to explore the mechanism of these results and further understand the physical and physiological effects of chronic insufficient sleep on lactating mothers.

Our present study has several limitations. First, the study's sample size is relatively small and only included Chinese mothers, which may lead to poor adaptability of the research results. Second, the mother's sleep duration was measured through self-reporting, not an objective indicator. Third, sleep quality was not explored in this study and should be investigated in subsequent research. In addition, this study did not investigate more information such as family income, family support, work status and education level, which may cause limitations in interpreting the results of this study. Finally, most mothers in this study stopped breastfeeding after the second milk collection, which hindered us from analyzing the relationship between more prolonged breastfeeding and sleep duration. Therefore, future studies can conduct similar investigations using larger sample sizes, multiple regions, and adopting objective indicators. Effective measures should be taken to improve the sleep duration of mothers during breastfeeding.

The findings of this study add to the existing body of knowledge about breast milk. In hindsight, the advantages of breastfeeding for maternal sleep deserve more attention. In addition to promoting the vital role of breastfeeding in the physiology of mothers and infants, more focus should be given to promoting the benefits of breastfeeding on maternal sleep. More research and greater emphasis on sleep can help encourage more mothers to breastfeed, prolong the breastfeeding period, and improve the quality of life of lactating mothers.

## Conclusions

In this study, it is common for lactating mothers to have insufficient sleep after delivery. We explored the relationship between maternal sleep duration and infants' feeding patterns. Our results show that formula feeding adversely affects sleep length among lactating mothers. We investigated the relationship between sleep duration and milk macronutrients of lactating mothers and found no significant correlation between sleep duration and milk macronutrients. When analyzing the relationship between sleep duration and related hormones, we found a positive correlation between plasma GHRL and sleep duration among lactating women. More high-quality studies are needed to clarify the mechanisms of these findings to provide a solid theoretical basis and support references for breastfeeding.

## Data availability statement

The original contributions presented in the study are included in the article/supplementary materials, further inquiries can be directed to the corresponding author.

## Ethics statement

The studies involving human participants were reviewed and approved by the Ethics Committee of the Xinhua Hospital with approval number XHEC-C-2020-081. The patients/participants provided their written informed consent to participate in this study.

## Author contributions

HR, WC, and QT contributed to the conception of the study. HR, YZ, XuaZ, XueZ, YX, and WG performed the experiment. HR contributed significantly to analysis, manuscript preparation, performed the data analysis, and wrote the manuscript. QT, YZ, YF, and WC helped perform the analysis with constructive discussions. All authors contributed to the article and approved the submitted version.

## Funding

This study was supported by grants from the Shanghai Key Laboratory of Pediatric Gastroenterology and Nutrition (17dz2272000), the Foundation of Shanghai Municipal Health Commission (Key weak discipline construction project 2019ZB0101), and the Scientific Research Fund of China Nutrition Society (CNS-HPNK2021-16).

## Conflict of interest

The authors declare that the research was conducted in the absence of any commercial or financial relationships that could be construed as a potential conflict of interest.

## Publisher's note

All claims expressed in this article are solely those of the authors and do not necessarily represent those of their affiliated organizations, or those of the publisher, the editors and the reviewers. Any product that may be evaluated in this article, or claim that may be made by its manufacturer, is not guaranteed or endorsed by the publisher.
